# LIM1 contributes to the malignant potential of endometrial cancer

**DOI:** 10.3389/fonc.2023.1082441

**Published:** 2023-03-10

**Authors:** Hiroaki Kato, Noritaka Saeki, Matome Imai, Hiroshi Onji, Akiko Yano, Shuhei Yoshida, Tomohisa Sakaue, Toru Fujioka, Takashi Sugiyama, Yuuki Imai

**Affiliations:** ^1^ Department of Obstetrics & Gynecology, Ehime University Graduate School of Medicine, Toon, Ehime, Japan; ^2^ Division of Medical Research Support, Advanced Research Support Center, Ehime University, Toon, Ehime, Japan; ^3^ Division of Integrative Pathophysiology, Proteo-Science Center, Ehime University, Toon, Ehime, Japan; ^4^ Department of Pathophysiology, Ehime University Graduate School of Medicine, Toon, Ehime, Japan; ^5^ Department of Cardiovascular and Thoracic Surgery, Ehime University Graduate School of Medicine, Toon, Ehime, Japan; ^6^ Division of Cell Growth and Tumor Regulation, Proteo-Science Center, Toon, Ehime, Japan

**Keywords:** endometrial cancer, Lim1, knockdown, CREB signaling, RNA-sequencing, ingenuity pathway analysis

## Abstract

**Introduction:**

The incidence of endometrial cancer (EC) has been increasing worldwide. However, because there are limited chemotherapeutic options for the treatment of EC, the prognosis of advanced-stage EC is poor.

**Methods:**

Gene expression profile datasets for EC cases registered in The Cancer Genome Atlas (TCGA) was reanalyzed. Highly expressed genes in advanced-stage EC (110 cases) compared with early-stage EC (255 cases) were extracted and Gene Ontology (GO) enrichment analysis was performed. Among the enriched genes, Kaplan-Meier (KM) plotter analysis was performed. Candidate genes expression was analyzed in HEC50B cells and Ishikawa cells by RT-qPCR. In HEC50B cells, LIM homeobox1 (LIM1) was knocked down (KD) and cell proliferation, migration, and invasion ability of the cells were evaluated. Xenografts were generated using LIM1-KD cells and tumor growth was evaluated. Ingenuity Pathway Analysis (IPA) of RNA-seq data using LIM-KD cells was performed. Expression of phospho-CREB and CREB-related proteins were evaluated in LIM1-KD cells by western blotting and in xenograft tissue by immunofluorescent staining. Two different CREB inhibitors were treated in HEC50B and cell proliferation was evaluated by MTT assay.

**Results:**

Reanalysis of TCGA followed by GO enrichment analysis revealed that homeobox genes were highly expressed in advanced-stage EC. Among the identified genes, KM plotter analysis showed that high LIM1 expression was associated with a significantly poorer prognosis in EC. Additionally, LIM1 expression was significantly higher in high-grade EC cell lines, HEC50B cells than Ishikawa cells. Knockdown of LIM1 showed reduced cell proliferation, migration and invasion in HEC50B cells. Xenograft experiments revealed that tumor growth was significantly suppressed in LIM1-KD cells. IPA of RNA-seq data using LIM-KD cells predicted that the mRNA expression of CREB signaling-related genes was suppressed. Indeed, phosphorylation of CREB was decreased in LIM1-KD cells and LIM1-KD cells derived tumors. HEC50B cells treated by CREB inhibitors showed suppression of cell proliferation.

**Conclusion and discussion:**

Collectively, these results suggested that high LIM1 expression contributed to tumor growth *via* CREB signaling in EC. Inhibition of LIM1 or its downstream molecules would be new therapeutic strategies for EC.

## Introduction

The incidence of endometrial cancer (EC) has been increasing worldwide for patients of all ages (1). Because patients frequently recognize symptoms of abnormal genital bleeding during early-stage EC, surgical treatment is typically performed early and is effective. However, advanced-stage EC and recurrent cases require adjuvant treatment, such as chemotherapy and radiation therapy, in addition to surgery, and the prognosis of these patients remains poor (2). Although treatments with immune checkpoint inhibitors (3) and multikinase inhibitors (4) have recently been applied for recurrent and refractory EC, the options for chemotherapy and molecular-targeted therapy are limited for patients with advanced-stage EC compared with those for patients with other types of cancers, such as hematologic cancers (5); therefore, novel therapeutic targets and drugs for EC are urgently required. Recently, integrative genome-wide analyses have been widely utilized to identify target molecules for cancers by exploiting publicly available datasets registered in databases, such as The Cancer Genome Atlas (TCGA) (6) and Gene Expression Omnibus (GEO) (7). For example, we have identified *GPRC5A* as a possible therapeutic target for prostate cancer using integrative genome-wide analyses with nonbiased datasets (8). In this study, in order to identify possible therapeutic targets for advanced EC, gene expression profiling datasets of publicly available endometrial uterine cancer cases registered in TCGA were analyzed. Integrative genome-wide analyses found that *HOMEOBOX* genes were significantly enriched in patients with advanced EC. Among these genes, LIM homeobox1 (LIM1) was found to be associated with a poor 5-year survival rate in patients with advanced EC. LIM1 knockdown (KD) in EC cell lines resulted in decreased cell proliferation and invasion. RNA-seq analysis demonstrated that LIM1 upregulates CREB signaling-related genes and that LIM1 KD led to reduced phosphorylation of CREB *in vitro* and *in vivo*, suggesting that LIM1 contributes to the malignancy of EC *via* CREB signaling.

## Material and method

### RNA-seq data analysis from TCGA

To identify candidate genes as new therapeutic targets for EC, we re-analyzed publicly available databases. RNA-seq data were obtained from the Genomic Data Commons DATA Portal (https://gdc.cancer.gov/) and analyzed independently. mRNA expression data normalized by the fragments per kilobase of exon per million mapped fragments (FPKM) method were extracted from TCGA (FIGO stage I: 255 samples, stages II–IV: 110 samples). FPKM values were compared between stage I and stages II–IV, and genes that showed more than 2-fold differences in expression in the stages II–IV group on average and significant differences by Student’s *t*-tests were designated as differentially expressed genes (DEGs). DEGs were then subjected to Gene Ontology (GO) enrichment analysis using Database for Annotation, Visualization and Integrated Discovery (DAVID) Bioinformatics Resources 6.8 (9, 10).

### Kaplan-Meier plot analysis

Kaplan-Meier plot analysis was conducted using Kaplan-Meier plotter software (http://kmplot.com/analysis/index.php?p=service&cancer=pancancer_rnaseq) (11, 12).

### Cell culture

Ishikawa and HEC50B human EC cells were obtained from the Japanese Collection of Research Bioresources Cell Bank. EC cells were cultured in Dulbecco’s modified Eagle medium (DMEM) with heat-inactivated 10% fetal bovine serum (FBS; MP Biochemicals, New Zealand) and 1% antibiotics (100× diluted antibiotic-antimycotic; Gibco, CA, USA) at 37°C in a humidified atmosphere containing 5% CO_2_.

### Retrovirus transduction

A Platinum Retrovirus Expression System (Amphotropic, VPK-301; Cell Biolabs, USA) was used to overexpress LIM1 in EC cell lines. Packaging cells were cultured in DMEM containing 10% FBS, 1 μg/mL puromycin, 10 μg/mL blasticidin, and 10 μg/mL penicillin/streptomycin. The cDNA sequence of *LIM1* was incorporated into the pMx-vector (pMx-LIM1-vector), and the pMx-LIM1-vector was transfected into packaging cells by lipofection using Lipofectamine 3000 (Thermo Fisher Scientific, USA). Twenty-four hours after lipofection, the medium was replaced with antibiotic-free medium followed by additional incubation for 24 h. After filtration of the virus-containing supernatant using a polyvinylidene difluoride (PVDF) membrane with a 0.22-μm pore size (Merck Millipore, Ireland), Ishikawa cells were cultured in the filtered supernatant supplemented with 8 μg/mL polybrene (hexadimethrinebromide; cat. no. H9268-5G; Sigma Aldrich, St. Louis, MO, USA). After 4 h, antibiotic-free DMEM was added to maintain the concentration of polybrene at less than 4 µg/mL. After 24 h, the medium was replaced with DMEM supplemented with 10 μg/mL puromycin.

### Lentiviral transduction

Trans-Lentiviral shRNA Packaging mix (cat. no. TLP5912) and lentivirus vector encoding both LIM1 shRNA and green fluorescent protein (GFP; SMART vector Lentiviral Controls, SMART vector Lentiviral shRNA: Reporter: TurboGFP promotor: hCMV targeted region: ORF- V3SH11240-230903623=KD#1; Reporter: TurboGFP promotor: hCMV targeted region: 3’UTR- V3SH11240-225324807=KD#2) were obtained from horizon (England). Each shRNA vector and packaging vector were cotransfected into HEK293T cells using Lipofectamine 3000 (Thermo Fisher Scientific) according to the manufacturer’s instructions. To establish HEC50B sublines stably expressing *LIM1* shRNA and control vector (LIM1-KD and control cells, respectively), lipofection of each virus vector and lentivirus packaging mix into HEK293 cells was carried out using Lipofectamine 3000 (Thermo Fisher Scientific) according to the manufacturer’s protocol. After 24 h, the medium was changed to antibiotic-free DMEM, and cells were cultured for 48 h. After filtration of virus-containing supernatants using PVDF membranes with a 0.22-μm pore size (Merck Millipore), HEC50B cells were cultured in the viral supernatant supplemented with 8 μg/mL polybrene. After 4 h, antibiotic-free DMEM was added to maintain the concentration of polybrene to less than 4 µg/mL. After 24 h, the medium was replaced with DMEM supplemented with 10 μg/mL puromycin. The cells were then sorted based on GFP intensity using a FACSAria instrument (BD Biosciences, USA), and only cells with relatively strong GFP fluorescence were collected and used for subsequent experiments.

### Reverse transcription quantitative polymerase chain reaction

Total RNA was extracted using ISOGEN (Nippon Gene, Japan) and an RNeasy spin column kit (Qiagen, Germantown, MD, USA). cDNA was synthesized from 500 ng total RNA using Prime Script RT Master Mix (Takara Bio Inc., Japan). RT-qPCR was performed using SYBR Premix Ex Taq II (Takara Bio Inc.) with Thermal Cycler Dice (Takara Bio Inc.) according to the manufacturer’s instructions. Gene expression was normalized to that of *RPLP0* as a housekeeping gene. Primer sequences are listed in the **Supplemental Materials**.

### Cell counting assay

EC cell lines were seeded at 5 × 10^4^ cells/35 mmø dish and precultured for 24 h under FBS-starved conditions. At 24, 72, and 120 h after the medium was changed to DMEM containing FBS and antibiotics, the number of cells was counted using a TC20 cell counter (Bio-Rab Laboratories, Hercules, CA, USA). Results were normalized to the cell count at 24 h (t1).

### MTT assay

MTT (3-(4,5-dimethyl-2-thiazolyl)-2,5-diphenyltetrazolium bromide) assay kit (Nacalai Tesque, Japan) was used according to the manufacturer’s instructions. 1×10^3^ HEC50B cells in DMEM containing FBS were seeded to each well of 96 well plate. After 24 hours, the medium was replaced to DMEM containing FBS supplemented with CREB inhibitor, KG501 (Selleck, USA) or compoud3i (Selleck, USA), at concentrations of 0.5, 1, 5 and 10 μM. As vehicle control, DMSO was supplemented with DMEM. After 24, 48, 72 and 96 hours, HEC50B cells were incubated in medium with 0.5 mg/mL MTT solution for 2 hours and lysed with 0.04 M HCl in isopropyl alcohol. Absorbance at 570 nm was measured using Multiskan SkyHigh Microplate Reader (Thermo Fisher Scientific).

### Migration and invasion assays

HEC50B sublines (control and LIM1-KD cells) were precultured for 24 h under FBS-starved conditions. For migration assays, 4 × 10^5^ cells in serum-free DMEM were seeded in the upper chambers of 24-well Transwell inserts (8-μm pore size, polycarbonate membrane; Corning, USA), and complete medium containing 10% FBS was added to the lower chambers. After 24 h, cells were fixed, and the cells remaining on the upper membranes were removed using cotton swabs. Then, the cells that had migrated to the lower membranes were stained with Diff-quick (Sysmex, Japan) according to the manufacturer’s instructions. Cell numbers were counted under a microscope. For invasion assays, we used invasion Transwells manufactured by Corning Inc. (BioCoat Matrigel Invasion Chamber, 24-well plate, 8-μm pore size; cat. no. 354480; Corning). According to the manufacturer’s protocol, the chambers were hydrated before use, and cells were seeded as in the migration assays. Cells were incubated for 72 h and stained as described for migration assays, and the number of cells that passed through the membrane was counted.

### Western blotting

EC cell lines and xenograft tissues were lysed in RIPA buffer (Fujifilm Wako, Japan) containing a cocktail of proteases and phosphatase inhibitors (Nacalai Tesque, Inc., Japan) on ice. Following lysis, samples were sonicated and centrifuged at 20600 rpm for 10 min at 4°C, and supernatants were collected. 20 micrograms of the extract was mixed with sodium dodecyl sulfate (SDS) sample buffer and boiled at 100°C for 5 min. Samples were then applied to 10% SuperSep Ace polyacrylamide gels (Fujifilm Wako) and subjected to SDS-polyacrylamide gel electrophoresis. Proteins were transferred to PVDF membranes (Bio-Rad Laboratories) using a mini transblot cell (Bio-Rad Laboratories). The membranes were then blocked with Blocking One-P solution (Nacalai Tesque, Inc.) for 40 min at room temperature. The membranes were reacted with primary antibodies, including anti-LIM1 (R&D Systems, USA; cat. no. MAB2725; 1:5000 dilution), anti-glyceraldehyde 3-phosphate dehydrogenase (GAPDH; Cell Signaling Technology, USA; cat. no. 5174s; 1:5000 dilution), anti-CREB (Cell Signaling Technology; cat. no. 9197s; 1:5000 dilution), anti-phospho-CREB (Cell Signaling Technology; cat. no. 9198s; 1:5000 dilution), anti-extracellular signal-regulated kinase (ERK; Cell Signaling Technology; cat. no. 4695s; 1:5000 dilution), anti-phospho-ERK (Cell Signaling Technology; cat. no. 4370s; 1:5000 dilution) and anti-CREB Binding Protein (CBP; SANTA CRUZ; cat.no. sc-7300; 1:5000) at 4°C for 24 h. After washing with TBS containing 0.05% Tween20, membranes were incubated with secondary antibodies, including horseradish peroxidase (HRP)-conjugated anti-rabbit IgG (Dako, USA; 0.05 μg/mL) and HRP-conjugated anti-mouse IgG (Promega, Madison, WI, USA; 0.2 μg/mL), for 60 min at room temperature. ECL Prime Western Blotting Detection Reagent (Amersham, UK) was used to detect signals, and membranes were imaged using an Image Quant LAS4010 instrument (Cytiva).

### Immunoprecipitation

Cells were lysed in ice-cold Pierce™ IP Lysis Buffer (Thermo Fisher Scientific) with gentle pipetting and then incubated on ice for 30 minutes. The lysate was vortexed followed by centrifugation at 4°C. The supernatant was collected and used as a sample for immunoprecipitation. 1.5 mg of protein was mixed with 6 μg of anti-CREB Binding Protein (CBP; SANTA CRUZ; cat.no. sc-7300) and rotated slowly at 4°C for overnight. 1500 μg of Dynabeads Protein G (Thermo Fisher Scientific) was added to the protein-antibody complex and then the mixture was slowly rotated for 30 minutes at 4°C. After 2 times washing of beads, Dynabeads were dissolved in RIPA buffer. Obtained protein lysates were used for western blotting.

### Animals

BALB/cAJcl-nu nude female mice were obtained from CLEA Japan. Mice were housed in a specific-pathogen-free facility under climate-controlled conditions with a 12-h light/dark cycle and were provided water and a standard diet (MF; Oriental Yeast, Japan) *ad libitum*. Animal experiments were conducted with the permission of the Animal Experiment Committee of Ehime University (approval no. 37A12-16) and were performed in accordance with Ehime University Guidelines for Animal Experiments.

### Tumor xenografts

HEC50B sublines were suspended in a mixture of DMEM and Matrigel solution (Corning, USA; 1:12, 1 × 10^5^ cells/mL). One hundred microliters of cell suspension was inoculated subcutaneously into 6-week-old BALB/cAJcl-nu nude female mice. Tumor volumes were measured once per week using vernier calipers and calculated using the following formula: tumor volume = (length × width^2^)/2. Tumor weights were measured at the end of the experiment (6 weeks).

### RNA-seq and data analysis

Total RNA samples were obtained from HEC50B sublines using RNeasy spin column kits and verified using an Agilent 2100 Bioanalyzer. RNA-seq was performed on an Illumina NextSeq 500 with a read configuration of 75 bp for single reads; 15 million reads were generated per sample. Mapping of trimmed fastq files to the human hg19 dataset was performed with Hisat2 (Unix program). Then, the number of reads was counted using featureCounts and normalized with the tmm method. DEGs were detected with a sensitivity of less than 0.05 for the false discovery rate (FDR). Data were registered in the GEO with accession number GSE215413.

### Immunocytochemical staining

EC cells were fixed with 4% paraformaldehyde for 5 minutes and then permeabilized for 10 min with 0.5% Triton X in phosphate-buffered saline (PBS) and blocked by treatment with 1% bovine serum albumin in 0.02% Triton X-PBS. Primary antibodies were added at 1:100 (anti-LIM1) and incubated for 1 h at room temperature. After washing, secondary antibodies (AlexaFluor 568 mouse IgG, 1:1000 dilution) mixed with AlexaFluor 488-conjugated phalloidin (1:40) and 5 μg/mL 4′,6-diamidino-2-phenylindole (DAPI) were added, and samples were incubated for 30 min at room temperature.

### Histological analysis

Xenograft tissues were fixed with 4% paraformaldehyde for 4 h and then embedded in paraffin. Sections were then cut to 3–4 μm thickness. For hematoxylin-eosin (HE) staining, deparaffinized sections were stained with Carazzi’s hematoxylin for 10 min, followed by staining with eosin Y for 10 min. For immunofluorescent staining, deparaffinized sections were boiled in ImmunoSaver (Fujifilm Wako) at 98°C for 60 min for antigen retrieval. After blocking with Blocking One Histo (Nacalai Tesque, Inc.) for 60 min, sections were incubated with primary antibodies, including anti-human vimentin (Invitrogen, Carlsbad, CA, USA; cat. no. MA5-11883; 1:50 dilution) and anti-phospho-CREB (Cell Signaling Technology; 1:200 dilution), diluted in Signal Enhancer HIKARI (Immunostain Solution A; Nacalai Tesque, Inc.) overnight at 4°C. After washing, sections were incubated with secondary antibodies (AlexaFluor 568-conjugated goat anti-rabbit IgG [Thermo Fisher Scientific; 1:400 dilution] and AlexaFluor 488-conjugated goat anti-mouse IgG; 1:400 dilution]) and DAPI (5 μg/mL) diluted in Signal Enhancer HIKARI (Immunostain Solution B; Nacalai Tesque, Inc.) for 60 min at room temperature.

### Statistical analysis

Two-tailed unpaired Student’s *t*-tests and Mann-Whitney U tests were performed using Prism 8 (GraphPad Software, USA) to analyze differences between two groups. Analysis of variance (ANOVA) followed by *post-hoc* Tukey’s tests were performed using Prism 8 (GraphPad Software) and SPSS (IBM, USA) to compare multiple groups. For all graphs, data are represented as means ± standard deviations. Statistical significance was accepted when the *P* value was less than 0.05.

## Results

**Figure 1:**
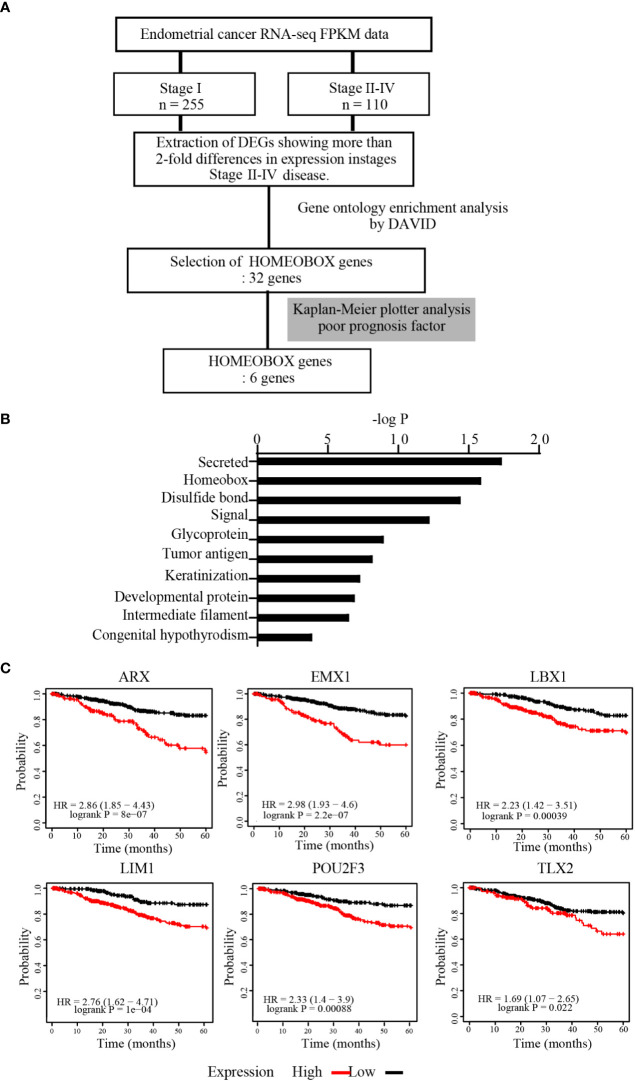
*HOMEOBOX* genes were enriched in upregulated genes in advanced EC. **(A)** Strategy for identification of upregulated genes from datasets registered in TCGA. **(B)** Gene Ontology (GO) enrichment analysis of genes extracted from TCGA that were upregulated more than 2-fold in stages II–IV EC compared with stage I EC by DAVID Bioinformatics Resources. **(C)** Analysis of the 5-year overall survival rate by Kaplan-Meier plotter for 6 genes within the *HOMEOBOX* gene cluster.

From TCGA, FPKM datasets for patients with stage I (n = 255) or stages II–IV (n = 110) EC were obtained. The mean FPKM values were then calculated for each molecule, and only those with a 2-fold or greater increase in expression in stages II–IV compared with stage I were selected for GO enrichment analysis by DAVID to identify the gene characteristics (**Figure 1A**). GO enrichment analysis revealed that several clusters were enriched, including “Secreted,” “Homeobox,” and “Disulfide bond” (**Figure 1B**). Among these genes, we focused on the *HOMEOBOX* gene group because Homeobox transcription factors can govern cell features. Among 32 genes included in the *HOMEOBOX* gene cluster, Kaplan-Meier plotter analysis revealed that upregulation of six genes (*ARX*, *EMX1*, *LBX1*, *LIM1*, *POU2F3*, and *TLX2*) was associated with significantly decreased 5-year survival rates in patients with EC (**Figure 1C**).

**Figure 2:**
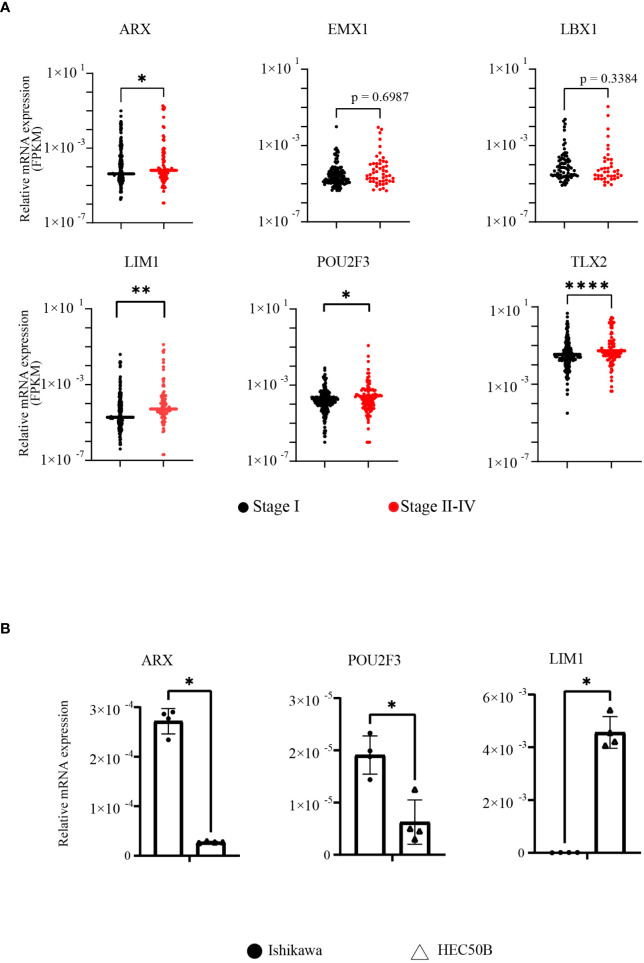
LIM1 was highly expressed in advanced EC and grade 3 EC cells. **(A)** Comparison of FPKM values between early- and advanced-stage EC groups in genes included in the *HOMEOBOX* group (stage I: n = 255, stages II–IV: n = 110, Mann-Whitney *U*-test). **(B)** Comparison of *HOMEOBOX* gene expression in EC cell lines by RT-qPCR. Ishikawa cells and HEC50B cells were derived from grade 1 and grade 3 disease, respectively (n = 3, Student’s *t*-test). *p < 0.05, **p < 0.01, ****p < 0.0001. Graphs show means ± standard deviations.

Next, FPKM data extracted from TCGA for each of the six genes were statistically analyzed to compare stage I and stages II–IV EC. The FPKM values of *ARX*, *LIM1*, *POU2F3*, and *TLX2* were significantly higher in patients with more advanced EC, although no significant difference was observed for *EMX1* and *LBX1* between the two groups (**Figure 2A**). In addition, in order to clarify the relationship between EC cell malignancy and expression levels for the 4 extracted genes, RT-qPCR was performed using total RNA extracted from EC cell lines representing two different disease grades, i.e., Ishikawa cells (grade 1) and HEC50B cells (grade 3); EC is divided into three grades, and higher grades are associated with worse prognoses. *TLX2* expression was not detected owing to nonspecific reaction of the primers. *ARX* and *POU2F3* expression levels were significantly lower in HEC50B cells than in Ishikawa cells. However, *LIM1* expression was significantly higher in HEC50B cells (**Figure 2B**). These results indicated that *LIM1* expression levels were positively correlated with advanced EC with poor prognosis and high-grade malignant EC, suggesting that LIM1 may be a therapeutic target for advanced EC.

**Figure 3:**
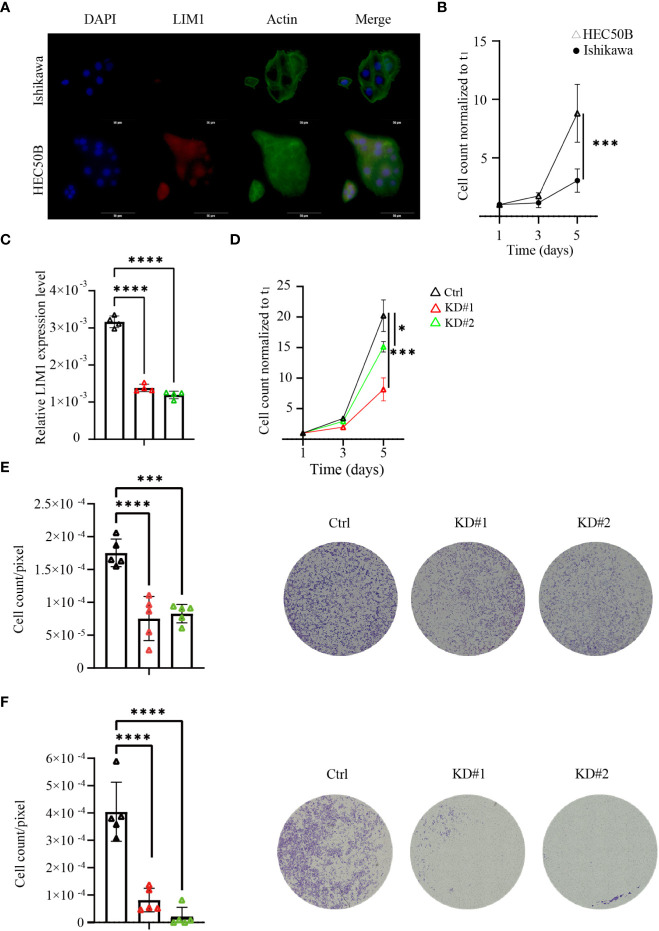
LIM1 knockdown (KD) in EC inhibited cell proliferation and invasion *in vitro*. **(A)** Immunocytochemical staining of LIM1 and phalloidin-actin staining in EC cells representing two different grades, i.e., Ishikawa cells and HEC50B cells. Red: LIM1, blue: nucleus, green: F-actin. Scale bar: 50 μm. **(B)** Cell counts to assess cell proliferation in Ishikawa cells and HEC50B cells (n = 3, Student’s *t-*tests). **(C)** RT-qPCR for *LIM1* using HEC50B cells treated with shControl (Ctrl) and shLIM1 (KD#1 and KD#2; n = 4, one-way ANOVA). **(D)** Cell counts to assess cell proliferation in Ctrl and KD sublines (n = 4, one-way ANOVA). **(E)** Migration assays using Transwells in Ctrl and KD sublines (n = 5, one-way ANOVA). Representative photographs are shown in the right panels. Scale bar: 50 μm. **(F)** Invasion assays using an extracellular matrix in Ctrl and KD sublines (n = 5, one-way ANOVA). Representative photographs are shown in the right panels. Scale bar: 50 μm. *p < 0.05, ***p < 0.001, ****p < 0.0001. Graphs show means ± standard deviations.

To validate whether LIM1 has biological potential to regulate malignancy in EC, we performed a comparison between Ishikawa cells and HEC50B cells. Immunofluorescence staining of these cell lines showed that LIM1 expression was clearly localized to the nucleus in HEC50B cells but not in Ishikawa cells, consistent with the RT-qPCR results (**Figure 3A**). In cell counting assays, proliferative potential was significantly higher in HEC50B cells than in Ishikawa cells, similar to previous reports (**Figure 3B**) (13). Next, LIM1-KD sublines were generated by shRNA transduction using lentivirus in HEC50B cells. LIM1 expression was significantly suppressed in LIM1-KD cells compared with that in control cells (**Figure 3C**). Cell proliferation was significantly suppressed in LIM-KD cells compared with that in control cells (**Figure 3D**). Migration assays showed a significant decrease in the migrated cell counts of the LIM-KD cells. (**Figure 3E**); however, invasion assays showed a significant decrease in the number of invading cells in the LIM1-KD cells compared with the control cells (**Figure 3F**). Next, we overexpressed LIM1 in Ishikawa cells, as confirmed by RT-qPCR and western blotting (**Supplemental Figures 1A, B**). Cell proliferation assays showed no significant differences between control cells and LIM1-overexpressing cells (**Supplemental Figure 1C**). Migration assays also showed no significant differences following LIM1 overexpression in Ishikawa cells (**Supplemental Figure 1D**). These results suggested that LIM1 could contribute to the development of malignant phenotypes in EC, but that LIM1 overexpression alone was not sufficient to induce more malignant phenotypes in Ishikawa cells. Accordingly, LIM1 overexpression must be accompanied by changes in other partner molecules to promote malignancy.

**Figure 4:**
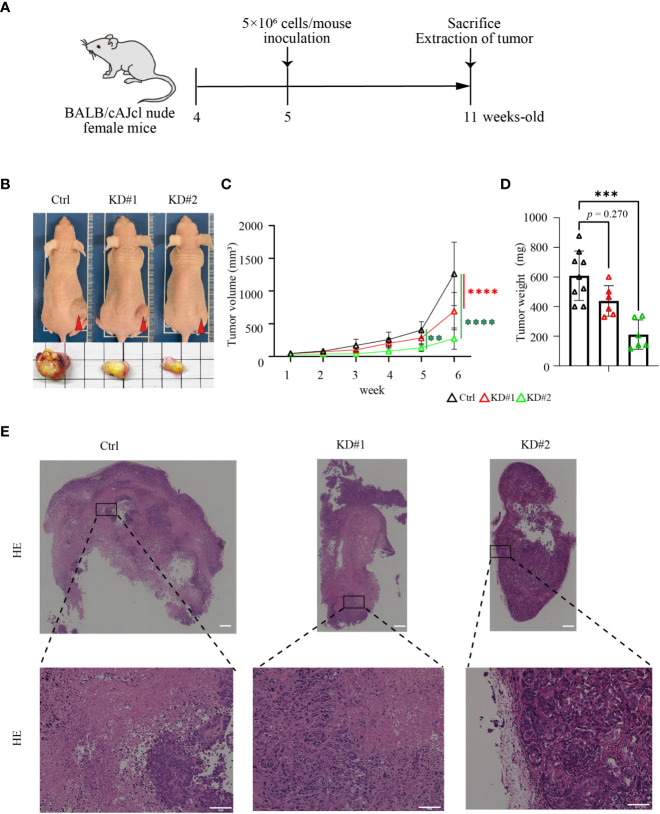
*LIM1* knockdown suppressed EC tumor growth *in vivo*. **(A)** Schematic of the xenograft model experiment. **(B)** Appearance of representative mice and excised tumors at 6 weeks after inoculation of HEC50B sublines. **(C)** Changes in the sizes of subcutaneous tumors over time (n = 6–9, one-way ANOVA). **(D)** Comparison of the weights of extracted tumors (n = 6–9, one-way ANOVA). **p < 0.01, ***p < 0.001, ****p < 0.0001. Graphs show means ± standard deviations. **(E)** HE staining of tumor tissues. Lower panels show high magnification of the boxed areas in upper panels. Scale bar: 50 μm.

To evaluate the function of LIM1 in tumor growth *in vivo*, xenograft experiments were performed using 4-week-old BALB/cAJcl nude female mice. After a 1-week adaptation period, control or LIM1-KD HEC50B cells were inoculated subcutaneously at 5 weeks of age, and tumors were removed after 6 weeks (**Figure 4A**). Subcutaneous tumors appeared relatively small for mice inoculated with LIM1-KD cells (**Figure 4B**), and the chronological size of tumors was significantly smaller in tumors of LIM1-KD cells than in those of control cells (**Figure 4C**). Evaluation of the weight of the excised tumors showed that LIM1-KD#1 tumors tended to be smaller, whereas LIM1-KD#2 tumors were significantly smaller when compared with control tumors (**Figure 4D**). HE staining of tumor sections revealed that the necrotic area was wide in control tumors but limited in LIM1-KD#1 tumors; LIM1-KD#2 tumor cells formed small clusters and were surrounded by connective tissue (**Figure 4E**). These results suggested that LIM1-KD tumors showed decreased cell proliferation and decreased invasive potential *in vivo*. These findings were consistent with the results of *in vitro* experiments, indicating that LIM1 was involved in tumor growth *in vivo*.

**Figure 5:**
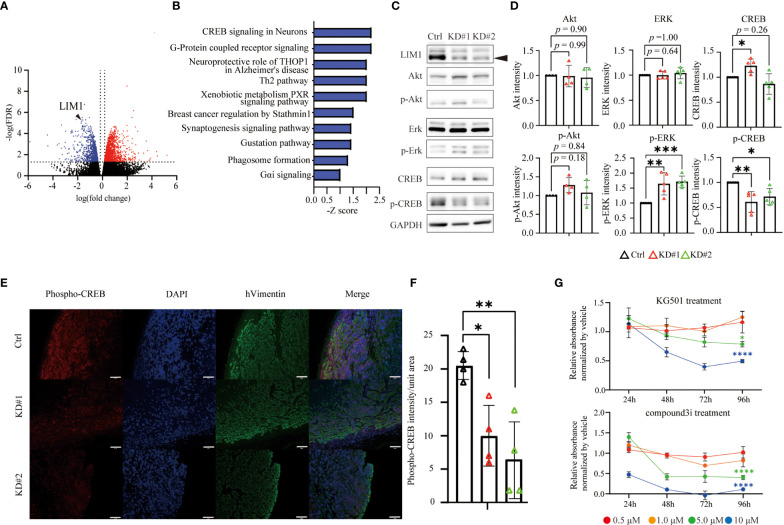
LIM1 promoted CREB phosphorylation in EC. **(A)** Volcano plot of DEGs in LIM1-KD (#1 and #2) cells versus Ctrl cells generated from RNA-seq results. **(B)** The highly ranked GO cluster among commonly downregulated genes in LIM1-KD cells compared with Ctrl cells by Ingenuity Pathway Analysis (IPA). **(C)** Western blotting of LIM1, ERK, phospho-ERK, CREB, phospho-CREB, and GAPDH using Ctrl cells and LIM1-KD cells. **(D)** Relative intensities of Akt, phospho-Akt, ERK, phospho-ERK, CREB, phospho-CREB bands by western blot analysis. Signals intensities were normalized to the GAPDH intensity in the same samples and then the intensity of the LIM1-KD (#1 and #2) cells were normalized to Ctrl cells [n = 5 (ERK, p-ERK, CREB, p-ERK), n = 4 (Akt, p-Akt) one-way ANOVA]. **(E)** Immunofluorescent staining for phospho-CREB and human vimentin in sections of xenograft tumors derived from Ctrl cells and LIM1-KD cells. Red: phosphorylated CREB, blue: nucleus, green: human vimentin. **(F)** Intensity of phospho-CREB in the tumor region (n = 4, one-way ANOVA). *p < 0.05, **p < 0.01. Graphs show means ± standard deviations. **(G)** MTT assay to assess cell proliferation. HEC50B cells were treated with CREB inhibitors, KG501 and compoun3i, respectively. Red, orange, green and blue indicate inhibitor concentrations at 0.5 μM, 1.0 μM, 5.0 μM and 10 μM, respectively. (n = 5, two-way ANOVA *p < 0.05, **p < 0.01, ***p < 0.001, ****p < 0.0001).

Finally, to explore the mechanisms underlying LIM1-mediated malignant phenotypes in EC cells, total RNA was extracted from control and LIM1-KD cells and subjected to RNA-seq. In the comparison of gene expression profiles, 567 genes were significantly upregulated, whereas 451 genes were significantly downregulated in both LIM1-KD#1 cells and LIM1-KD#2 cells compared with control cells (**Figure 5A**; FDR: 1.0, fold change > 1.5). Ingenuity Pathway Analysis (IPA) revealed that several pathways were commonly suppressed in LIM1-KD#1 cells and LIM1-KD#2 cells (**Figure 5B**). CREB signaling appeared near the top of the list and has been reported to contribute to cell proliferation together with cyclin D1 and signal transducer and activator of transcription 3 (14); therefore, we focused on CREB signaling. CREB signaling pathway-related genes were downregulated in LIM1-KD cells (**Supplemental Figure 2**). Therefore, we evaluated the activation of CREB and upstream molecules related to LIM1 expression levels and found that CREB phosphorylation was significantly suppressed and ERK phosphorylation was significantly upregulated in LIM1-KD cells. Total CREB expression was elevated in one of LIM1-KD subtypes (**Figures 5C, D**). In addition, to validate CREB activation by LIM1 *in vivo*, tumor tissue sections of xenografts derived from HEC50B sublines were evaluated by immunofluorescence staining. The signal intensity of phospho-CREB was evaluated in cell masses expressing vimentin as a tumor marker. The results showed that the signal intensity of phospho-CREB was significantly decreased in LIM1-KD tumors compared with that in control tumors (**Figures 5E, F**). In general, phosphorylation of CREB can induce recruitment of transcriptional coactivators such as CBP and p300 (15). To analyzed relationship between phospho-CREB and CBP dependent on LIM1 levels, we performed immunoprecipitation using anti-CBP antibody, followed by detection of CREB phosphorylation. As a result, the level of phosphorylated CREB protein cooperated with CBP was decreased in LIM1-KD cells (**Supplemental Figure 3**). Finally, we investigated whether functional inhibition of CREB in HEC50B cells can mimic phenotype of LIM1-KD. The treatment of different types of CREB inhibitors significantly suppressed cell growth in a concentration- and time-dependent manner associated with LIM1-KD cells (**Figure 5G**). Taken together, these results indicated that LIM1 contributed to tumor growth through CREB phosphorylation in EC, suggesting that LIM1 may be a novel therapeutic target in EC.

## Discussion

EC is often treated by surgery, resulting in a good prognosis for patients with early-stage disease. Additionally, chemotherapy is often applied as adjuvant therapy for advanced-stage or recurrent EC after surgery; however, the prognosis remains poor. Recently, lenvatinib and pembrolizumab were approved in Japan for the treatment of recurrent uterine cancer, although evidence-based chemotherapeutic options remain limited. We performed genome-wide analysis of uterine cancer and found that the homeobox genes including LIM1 may be a prognostic factor based on an unbiased comparison of data from early and advanced uterine cancers; this concept was explored using *in vitro* and *in vivo* experiments. Among candidate genes, ARX, POU2F3, TLX2 and LIM1 were derived as molecules contributing to the malignant transformation of endometrial cancer. However, mRNA levels of ARX, POU2F3 and TLX2 were not associated with tumor grade by comparison between different cell lines. This inconsistency might be caused by the difference of gene expression pattern between original tumor cells and cell lines, however the evaluation of expression levels of identified homeobox genes including LIM1 in patient specimens may provide new guidance for the existing morphological-based grading of endometrial cancer. Further detailed verifications are needed in the future. Recently, an *in vitro* study reported that LIM1 may be a prognostic marker for EC, suggesting that LIM1 contributes to tumor grade (16). In 2013, TCGA proposed a new prognostic classification of EC by comprehensive genomic analysis. Additionally, to apply the classification into clinical practice, Proactive Molecular Risk Classifier for Endometrial Cancer (ProMisE) based on mainly immunohistological analysis as an alternative instead of DNA sequencing method has been developed and stratified subgroups in EC. However, it is still controversial how to combine ProMisE evaluations based on molecular classifications and existing prognostic methods based on classical histological remarks such as invasion of lymph vessels and myometrial invasion. Given our results that LIM1 has functions related to EMT in EC, additional consideration of LIM1 expression levels in EC would progress for the ProMisE classification (17). EC is commonly diagnosed by non-invasive transvaginal ultrasound and biopsy of endometrial tissue as an invasive procedure. However, these tests are often refused by patients resulting delayed diagnosis. A recent report provided the availability of metabolomic signature of blood samples for prognosis assessment of EC (18). To reveal the association between metabolite variation and LIM1 expression might allow a more accurate and convenient EC-specific assessment for malignancy. LIM1 is a critical molecule contributing to the formation of the uterine body during embryonic development by stimulating WNT7A, HOX10, and HOX11 expression (19). In fact, mice with systemic *Lim1* knockout show dysplasia of the uterine body (20). In human cancer research, inhibition of LIM1 reduces tumor growth and migration in renal clear cell carcinoma *in vitro* and reduces metastatic potential *in vivo* (21). In human tissues, LIM1 is expressed in normal endometrium and EC cell lines; however, its role remains unclear (22). The progression of malignancy is correlated with dedifferentiation, i.e., regression into a developmental stage; thus, the uterine development essential factor LIM1 may have roles in EC malignant progression. The cAMP/protein kinase A/CREB signaling axis is a promising therapeutic target in various cancers. Activation of this signaling pathway can promote or suppress cancer progression, depending on the cancer type (23). In EC, the peptide hormone leptin promotes tumor growth *via* phosphorylation of ERK and CREB, followed by expression cyclin D1 (23). In this report, factors associated with uterine development during embryogenesis were found to be upregulated in EC cell lines. Although the detailed roles of LIM1 in CREB signaling are still unclear, our findings indicated that LIM1 was involved in the intermolecular network of CREB signaling and was associated with CREB phosphorylation in EC. Thus, a comprehensive investigation of LIM1-mediated transcriptional regulation, such as ChIP-seq, is required to improve our understanding of the roles of LIM1 in EC. However, our current study was limited by a lack of antibodies with high specificity against LIM1. Our western blotting results showed that knockdown of LIM1 suppressed phosphorylation of CREB in HEC50B cells. There are multiple pathways for CREB phosphorylation, and LIM1 knockdown predicted inhibition of Akt, ERK and CaM kinase IV pathways in IPA (**Supplemental Figure 2**). Previous report of the LIM1 function in renal clear cell carcinoma have shown that LIM1 knockdown doesn’t affect Akt phosphorylation (24), supporting the results of our study (**Figures 5C, D**). Besides, increased levels of phospho-ERK were found in LIM1-KD cells (**Figures 5C, D**), although the upregulation might be a compensatory response to the suppression of downstream CREB phosphorylation. Collectively, it suggested that at least Akt and ERK pathways were not involved in CREB phosphorylation dependent on LIM1. Further investigations are required to identify a direct pathway for CREB phosphorylation *via* LIM1. In addition to the signaling between LIM1 and phosphorylated CREB, LIM1-mediated therapeutic strategies for EC should be considered. IPA showed downregulation of Stathmin-related molecules following knockdown of LIM1 in HEC50B cells (**Figure 5B**). Stathmin has been shown to antagonize microtubule stabilization induced by paclitaxel, which is commonly applied as a chemotherapeutic agent in the treatment of cancer, suggesting that Stathmin may elicit resistance to chemotherapy in endometrial cancer (25). In terms of therapeutic, since LIM1 cooperates with other co-factors to form complexes for transcription as reported (26), a molecularly targeted drug against the complexes to inhibit transcription and against the formation of the complexes would be a possible strategy to endometrial cancer. In addition, downstream genes regulated by LIM1 also would be therapeutic targets. In conclusion, LIM1 was highly expressed and associated with poor prognosis in advanced EC, and knockdown of LIM1 ameliorated malignant phenotypes in high-grade EC cells by inhibiting the CREB signaling pathway. Inhibitors of LIM1 or its downstream signaling molecules may have applications in the treatment of advanced EC.

## Data availability statement

The datasets presented in this study can be found in online repositories. The names of the repository/repositories and accession number(s) can be found below: https://www.ncbi.nlm.nih.gov/geo/, GSE215413.

## Ethics statement

The animal study was reviewed and approved by the Animal Experiment Committee of Ehime University.

## Author contributions

HK, NS and SY was responsible for all experiments. TF, ToS and YI conceived the study ideas and advised on statistical analysis methods. NS contributed to the interpretation of the results. HK drafted the manuscript, which was revised by NS and YI. MI, HO, AY and TaS oversaw the study and YI and HK were responsible for the study design. All authors reviewed the manuscript and revised it critically for intellectual content. All authors contributed to the article and approved the submitted version.
