# Supplementary material for: LIM1 contributes to the malignant potential of endometrial cancer
Source: Front Oncol. 2023 Mar 10;13:1082441. doi: 10.3389/fonc.2023.1082441 (PMC10036843; doi:10.3389/fonc.2023.1082441)
Supplement: Supplementary file 1 [file DataSheet_1.pdf]

frontiers

Supplementary Material

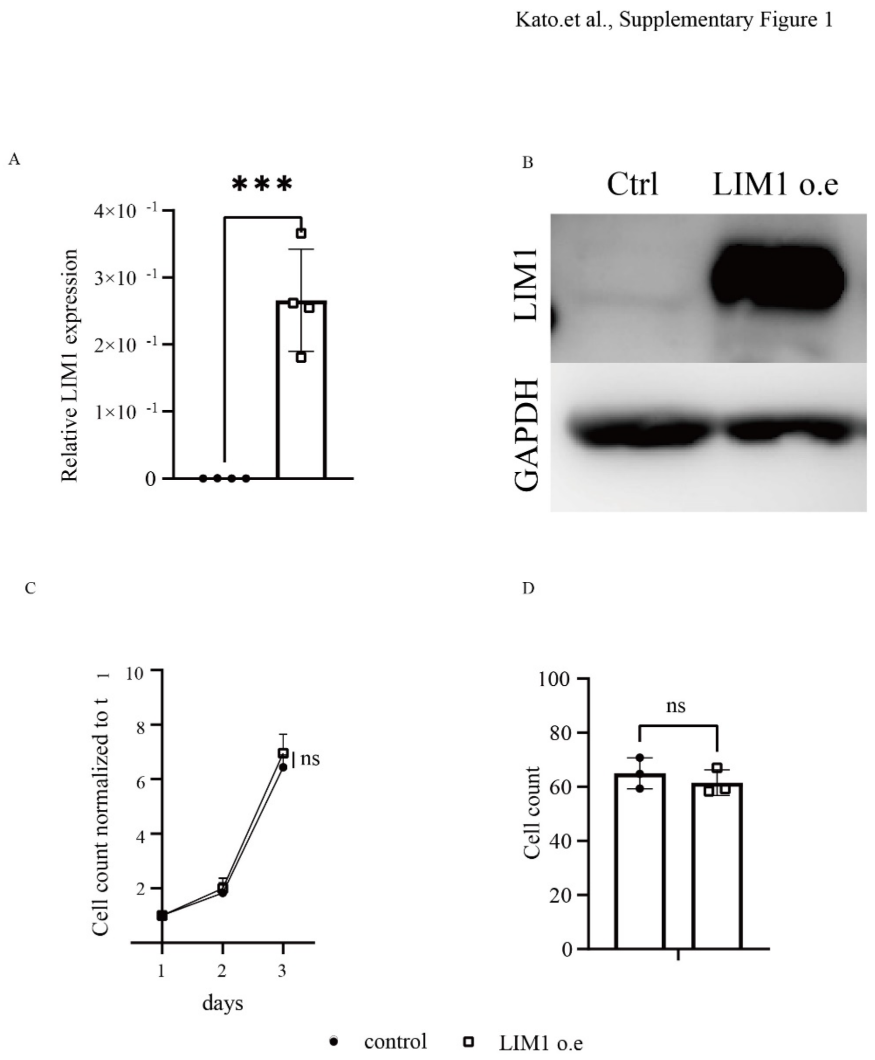

Supplementary Figure 1. Overexpression of LIM1 did not affect the phenotypes of Ishikawa cells.A RT-qPCR of LIM1 in Ishikawa cells with or without LIM1 overexpression (n = 4, Student's t-tests). B Western blotting for LIM1 using Ishikawa cells with or without LIM1 overexpression.C Cell counting assays to evaluate cell proliferation in Ishikawa cells with or without LIM1 overexpression (n = 4, Student's t-tests). D Migration assays using Transwell comparing Ishikawa cells with or without LIM1 overexpression (n = 4, Student's t-tests). ns: not significant, ***$p < 0.001$. Graphs show means ± standard deviations

Kato.et al., Supplementary Figure 2

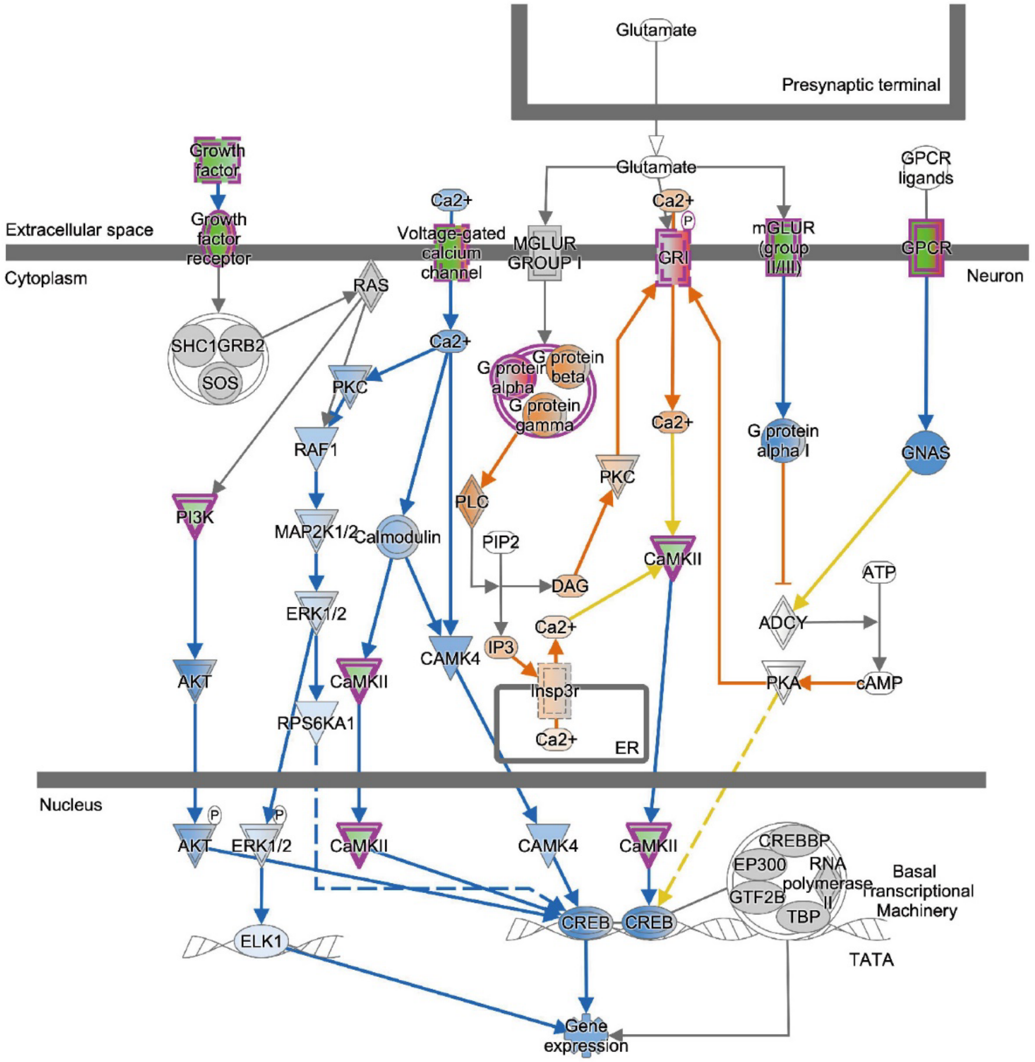

Supplementary Figure 2. Graphical depiction of the CREB signaling pathway.

The depiction was generated from RNA-seq data and Ingenuity Pathway Analysis (IPA) using DEGs obtained between Ctrl and LIM1-KD cells. Blue indicates downregulated molecules.

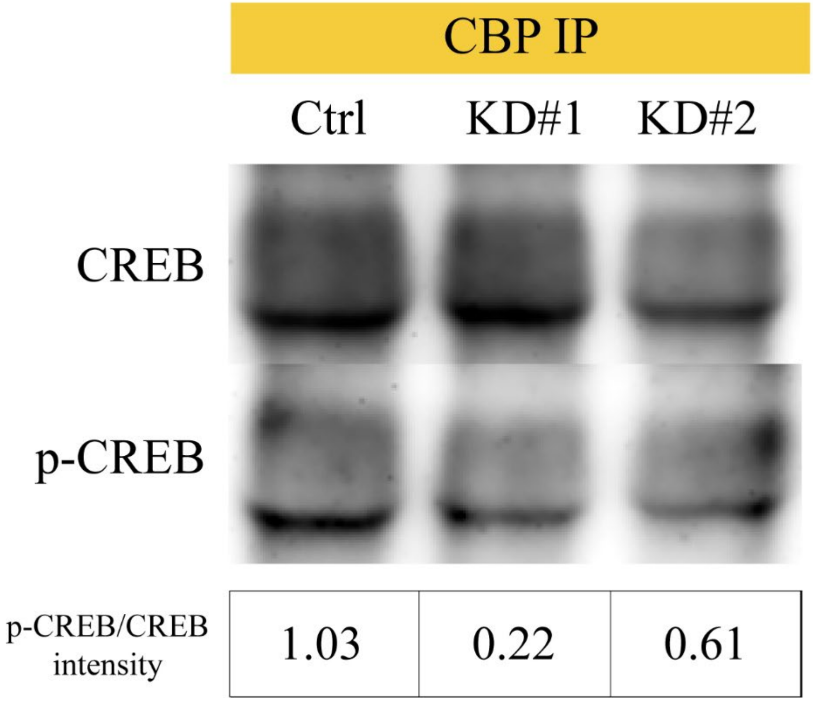

Supplementary Figure 3. Comparison of CREB phosphorylation by CBP IP in LIM1-KD cells.

After IP, western blotting was performed, then, detected and measured the band intensity using each antibody. Finally, the value of the intensity of pCREB was normalized by the value of the one of CREB.
